# Supplementary material for: P2X7 Receptor Modulation of the Gut Microbiota and the Inflammasome Determines the Severity of Toxoplasma gondii-Induced Ileitis
Source: Biomedicines. 2023 Feb 14;11(2):555. doi: 10.3390/biomedicines11020555 (PMC9952899; doi:10.3390/biomedicines11020555)
Supplement: Supplementary file 1 [file biomedicines-11-00555-s001.zip › biomedicines-2199971-supplementary.pdf]

Supplementary contents

1. **Supplementary Materials and Methods:**

1.1 Parasitic load in the ileum

Ileal samples were macerated and centrifugated at 250 x g for 5 minutes, and the pellet was resuspended in DMEM media and incubated with LLC-MK2 cells overnight at 37 °C in a humid atmosphere with 5% of the CO_2. After seven days, the cells were fixed and stained with fast panoptic, and the plaque forming was used to quantify the parasitic load. The parasite load was confirmed by semiquantitative PCR. The gDNA from ileum fragments was isolated using the Trizol reagent according to the manufactured instructions. The PCR was performed using the Gotaq green master mix, 2ug of DNA, and *Toxoplasma* β-actin (*Tg* β-actin) or murine β-actin primers. *Tg* β-actin gene- forward primer: GACCTTACCGAGTACATGATGAAG, reverse primer: CCATCGGGCAATTCATAGGAC; Ms β-actin gene- forward primer TATGCCAACACAGTGCTGTCTGG, reverse primer: TACTCCTGCTTGCTGATCCACAT with the following thermocycling program: denaturation 95 ºC for 1 minute, 40 cycles of annealing at 60 ºC for 1 minute, extension at 72 ºC for 5 minutes. The PCR products with *Tg* β-actin 185 bp, and murine β-actin 295 bp, were revealed in a 1.5% agarose gel stained with gel red.

1.2 Histologic analysis

Ileal samples were fixed immediately in 40 g/L formaldehyde saline for at least 24h and embedded in paraffin. The ileum was cut into 5μm sections, stained with hematoxylin and eosin (HE), and analyzed microscopically. To obtain the inflammatory score, histological parameters such as ulceration, hyperplasia, and inflammatory infiltrate were evaluated. The Paneth cells and granules number were measured based on the crypts of the ileum by HE stains. The cells and granules were counted per crypt in at least 10 different areas per tissue section under light microscopy, and the overall number of the cells and granules was expressed by percentage. The periodic acid of Schiff (PAS) technique was used to stain goblet cells within the ileal mucosa. The cells PAS-positive was counted in at least 500 epithelial cells in the crypts and surface epithelium by light microscopy, and the density of goblet cells was defined by percentage. Phosphomolybdic acid picrosirius red dye was used to stain collagen fibers in tissue. At least 5 different areas per tissue section were analyzed under light microscopy connected to a computer-assisted image analyzer.

1.3 Immunohistochemistry

Paraffin sections were cut onto slides pretreated with poly-lysine to characterize Paneth cells and intracellular signaling pathways using the indirect immunoperoxidase technique. Briefly, deparaffinized sections were first incubated at 90°C in 0.01 M sodium citrate buffer (pH 6.0) for 30 min for antigen retrieval. After endogenous peroxidase activity was blocked using 3% hydrogen peroxide in methanol for 30 min, the sections were rinsed in phosphate-buffered saline (PBS) and incubated in 5% bovine serum albumin (BSA) with 0.1% Triton for 30 min. Staining was carried out using the following primary antibodies diluted in 1% BSA: rabbit

For the indirect immunoperoxidase technique, paraffin sections were first cut onto slides pretreated with poly-lysine. After deparaffinization, sections were incubated at 90°C in 0.01 M sodium citrate buffer (pH 6.0) for 30 min for antigen retrieval. Then, the slides were immersed in 3% hydrogen peroxide in methanol for 10 min to block endogenous peroxidase activity. After being rinsed in phosphate-buffered saline (PBS) containing 0.5 % Tween 20 for 10 min, the tissue sections were incubated with non-immune horse serum for 30 min and, subsequently, with the appropriate antibody. Immunohistochemical staining was performed using the following primary antibodies: rabbit polyclonal anti-P2X7R (1:200) (Alomone Labs, Jerusalem, Israel); rabbit polyclonal anti-ki67 (1:200) (Merck KGaA, Darmstadt, Germany); rabbit monoclonal anti-CD4 antibody (1:50; Santa Cruz Biotechnology Inc., Santa Cruz, CA); rabbit monoclonal anti-CD11b antibody (1:100; ab133357); rabbit polyclonal anti-Caspase-1 antibody 1:500; ab1872); rabbit monoclonal anti-NLRP3 antibody (1:500; EPR23094-1) (all from Abcam, Cambridge,

United Kingdom). Two sections from each sample were incubated with either PBS alone or an isotype monoclonal IgG (concentration matched) and served as the negative controls. After incubation in a humidified chamber overnight at 4°C, the tissue sections were rinsed with PBS and incubated with a Dual Link System-HRP (Dako, Glostrup, Denmark) for 30 min at room temperature. Additional rinsing was followed by development with a solution containing hydrogen peroxide and diaminobenzidine (Dako, Glostrup, Denmark). The preparations were lightly counterstained in Harris's hematoxylin, dehydrated, and mounted in Permount (Fisher Scientific, Pittsburgh, PA, USA).

1.4 Assessment of apoptosis in the ileum

Apoptosis was assessed in ileal sections using a terminal deoxynucleotidyl transferase (TdT)-mediated dUTP nick-end labeling (TUNEL) assay. Samples from all experimental groups were analyzed using an ApopTag Peroxidase In Situ Apoptosis Detection Kit (Millipore Corporation, Billerica, MA, USA). First, paraffin sections were deparaffinized, hydrated, and incubated with a proteinase K solution. After endogenous peroxidase activity was blocked, the slides were covered with equilibration buffer and then incubated with a solution containing TdT enzyme. For the negative controls, we incubated a second section from each sample without the TdT enzyme. For the positive controls, we pretreated samples with DNase I (Sigma-Aldrich, Deisenhofen, Germany). After the reaction was terminated, the specimens were incubated with nonimmune horse serum and then incubated with an anti-digoxigenin antibody peroxidase conjugate. Development and counterstaining of sections were performed exactly as described above for immunohistochemistry. Morphologically preserved TUNEL-positive cells and apoptotic bodies were referred to as apoptotic cells.

1.5 Quantitative assessment of ileal sections

Quantitative analysis of tissue sections (under light microscopy) was carried out using a computer-assisted image analyzer (Leica QWin Plus V 3.5.1, Leica Microsystems, Ltd., Switzerland). Any epithelial and lamina propria cells exhibiting identifiable reactivity distinct from the background were regarded as positive. The results of the quantitative analysis of the cell subsets are expressed as the number of cells per mm^2 of longitudinally sectioned ileum tissues. Two independent observers who were unaware of the experimental data examined all tissue sections and captured images. To increase the resolving power of the microscope, some images were captured under oil immersion.

1.6 Myeloperoxidase Activity Assessment

Ileal samples were collected and frozen at −80◦C until the extraction of myeloperoxidase (MPO) when they were homogenized in potassium phosphate buffer (pH 6.0), frozen and defrosted twice, homogenized again in the potassium phosphate buffer (pH 6.0) containing 0.5% hexadecyl-trimethyl-ammonium bromide (Sigma Chemical Co., St. Louis, MO, USA), and centrifuged at 40,000 g for 30min at 4◦C. The supernatants were discarded, and the insoluble pellets were homogenized in the potassium phosphate buffer (pH 6.0) containing 0.5% hexadecyl-trimethyl-ammonium bromide. Ten microliters of the supernatants were added to a 96-well plate containing 290 µl of 50mM potassic PBS (pH 6.0), 3 µl of the substrate solution, containing 20 mg/ml o-dianisidine (Sigma Chemical Co., St. Louis, MO, USA), and 3 µl of H2O2 (20mM). The plate components were mixed, and the absorbance was determined at 460 nm for 1min with a spectrophotometer. MPO activity was measured by a standard curve of the samples in units of MPO/mg of colonic samples.

1.7 Nitric oxide production

Fresh ileum samples were collected, macerated in PBS containing 1% of protease inhibitor (Sigma-Aldrich, Darmstadt, St. Louis, MO, USA) on ice, and centrifuged at 1000 x g for 5 minutes. The supernatant was collected and subjected to the colorimetric assay using the Griess method to measure nitric oxide indirectly. Initially, in a 96-well plate, the sulfanilamide solution (1% sulfanilamide in 5% phosphoric acid, both of Sigma-Aldrich, St. Louis, USA) was added to all

experimental samples and wells containing the dilution series for the nitrite standard reference curve. Thereafter, the NED solution (0.1% N-1-naphthyl ethylenediamine dihydrochloride, Sigma-Aldrich, St. Louis, USA, in water) was dispensed into all wells. To ensure the accuracy of NO^{-2} quantification, the reference curve was prepared with the nitrite standard for each assay. The absorbance was measured within 30 min in a plate reader in 550 nm, read using the SpectraMax plate reader.

1.8 Analyzing of messenger RNA expression by qRT-PCR

Ileal fragments were processed to RNA extraction using Trizol reagent (Thermo Scientific, Wilmington, DE, USA) and cDNA construction using the High-capacity kit (Life Technologies, Carlsbad, CA, USA) according to the manufactured instructions. The RT-qPCR was performed using 1ug of DNA. The expression levels of selected genes were validated by quantitative real-time PCR (qRT-PCR). Real-time RT-PCR was performed with an ABI Prism 7500 (Applied Biosystems, Foster City, CA, USA) using a CustomTaqMan® Array Gene Signature Plate (Thermo Scientific, Wilmington, DE, USA), including the p2xr7 forward primer: ATGCCGGCTTGCTGCAGCTGGAACGATGTCTTTCAGTATGAGACA, reverse primer: CCAAGTCTTGTGAAAGGTACAAGAGATGTTCATACCTGGTAAGAT, nlrp3 forward primer: TGTGTGGATCTTTGCTGCG, reverse primer: GTTACTGTGCACATGTAGTGTGTAATAAGG; aim2 forward primer: TTGTCTCCTTCCTCGCACTT, reverse primer: TCGGGTAAGTGTTAACAAGG; nlrp6 forward primer: CCGTGTCCGAGTACAAGAAGAAC, reverse primer: CGCGATGAGCAGCTTGGT; caspase-11 forward primer: TGAAATGCATGTACTGAGAGCAAGG, reverse primer: CAATTGACTTGGGGATTCTG; caspase-1 forward primer: GGAAGCAATTTATCAACTCAGTG, reverse primer: GCCTTGTCCATAGCAGTAATG; and il-1β forward primer: TTCAGGCAGGCAGTATCACTC, reverse primer: CCACGGGAAAGACACAGGTAG genes. The mRNA levels were normalized to the expression levels of the control GAPDH forward primer: AGGTCGGTGTGAACGGATTTG, reverse primer: TGTAGACCATGTAGTTGAGGTCA gen. For the data analysis, the ΔΔCt method was used to determine the fold change of all of the target genes in each sample with 95 % confidence. The qRT-PCR reaction for each gene was performed in duplicate, and each experiment was repeated at least three times. The PCR cycles were performed according to the manufacturer's instructions.

1.9 Contractile activity of ileal longitudinal smooth muscle

At the end of the experimental period, on day 8, the animals were euthanized in a CO_2 chamber, and then segments of terminal ileum (1 cm long) were removed from C57BL/6 (WT) and $P2X7^{-/-}$ mice (2-3 months old). The longitudinal muscle strips were vertically mounted in a 5-ml organ bath filled with Krebs-Ringer solution (in mM; NaCl 118.3; KCl 4.7; $CaCl_2$ 2.5; $MgSO_2$ 1.2; KH_2PO_4 1.2; $NaHCO_3$ 25; glucose 11.1, pH 7,4) which was bubbled with carbogen (5% CO_2 and 95% O_2) at 37 °C. The tissues were equilibrated under a tension of 1 g (~ 10 mN) for 30 min until baseline tension was stable. At the end of the equilibration period, to test tissue viability, the ileum was contracted by adding KCl (100 mM) for ~5 min and 10 μM carbachol. Then the tissue was washed twice with the warm aerated Krebs solution at an interval of 15 min. Dose-response curves to acetylcholine (ACh) (0.01 – 30 μM) were constructed. The contractile responses were measured using an isometric force transducer and expressed as mN. Data were analyzed by nonlinear regression to estimate efficacy (E_{max}).

1.10 Fecal microbiota composition

Twenty-four samples (six per each experimental group) had their microbiome accessed from metabarcode analysis based on 16S rRNA, V3-V4 region [1]. The samples were extracted with the PureLink Microbiome DNA Purification Kit (Thermo Fisher Scientific, Waltham, MA, USA) according to the manufacturer’s instructions. The quality and quantity of the DNA were evaluated using a Nanodrop 2000 UV-vis Spectrophotometer (Thermo Scientific). Libraries were constructed using Nextera XT DNA Library Preparation kit (Illumina, California, U.S.A.) following the standard protocol. The quality of the libraries was measured using 2100 Bioanalyzer

Instrument. A pool was made with an aliquot of each sample. The samples were sequenced in the MiSeq System (Illumina, California, U.S.A.) and demultiplexed using by Illumina system.
The quality of the sequences was verified using MultiQC [2] . Samples were analyzed using Qiime2 (2019.7) platform (Boylen et al., 2019). The samples were denoised using the deblur extension, all amplicon sequence variants (ASVs) were aligned with Mafft, and the phylogeny was constructed using FastTree [3, 4]. Taxonomical annotation was made using vsearch and based on Silva v.123 database [5]. Barplots were constructed using the barplot extension, the alpha diversity was calculated based on Faith's Phylogenetic Diversity using alpha-group-significance extension, and the beta diversity was calculated based on weighted unifrac using the beta-group-significance extension [6, 7].

2. Supplementary Results:

2.1 Supplementary Figure S1

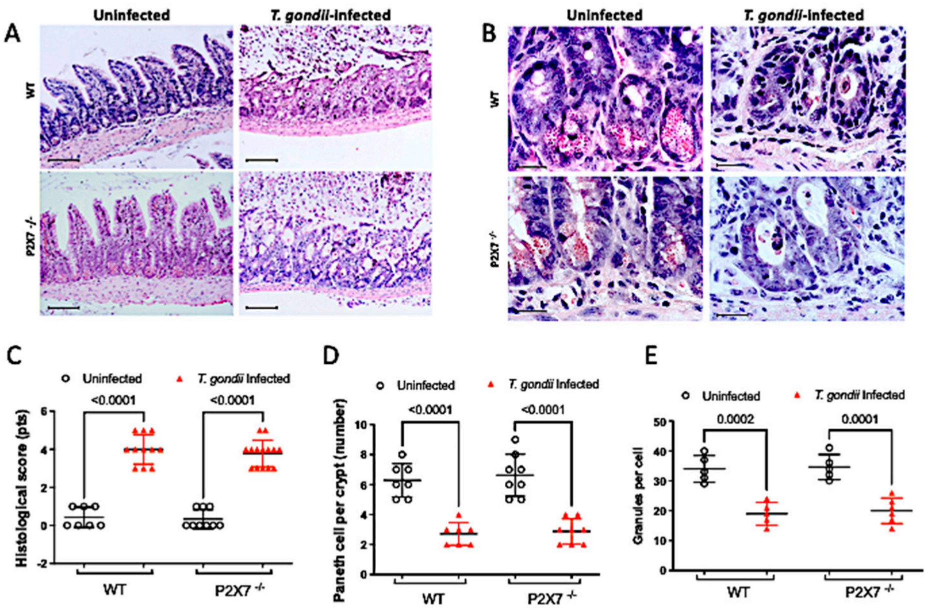

Supplementary Figure S1. *T. gondii* infection induces severe ileal inflammation and damage, with a significantly greater extension among the P2X7-/- mice. Histopathological analysis by hematoxylin and eosin (HE) staining of the terminal ileum shows severe inflammatory changes and tissue damage initiated by *T. gondii*-infection, but apparently not affected by the expression of the P2X7 (A, B). The density of Paneth cells and their granules showed a significant reduction in infected animals, but no change was attributed to the P2X7 expression (D, E). The scale bars represent 50µm. Data represent the means with SD of 7 to 12 animals per group. The analysis was performed by ANOVA, in which the post hoc Tukey test was used for multiple comparisons. Significant differences are presented.

2.1 Supplementary Figure S2

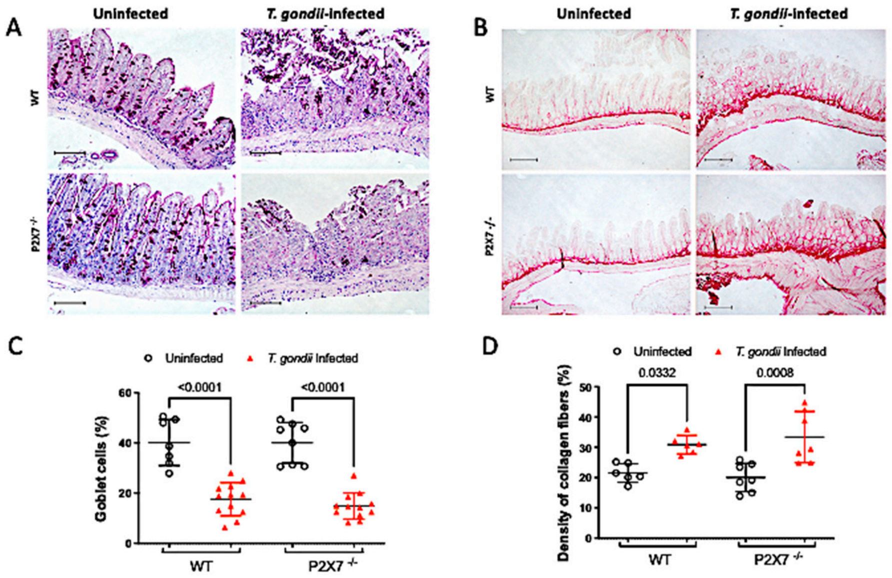

Supplementary Figure S2. *T. gondii* infection results in intense damage to specialized epithelial cells and deposition of collagen fibers in the ileum. Histopathological analysis revealed the increased loss of mucous-producing goblet cells labeled with periodic acid-Schiff (PAS) (A) and accumulation of collagen fibers labeled with picrosirius red dye (B) in *T. gondii*-infected mice. However, no difference was detected comparing WT with P2X7-/- mice (D, E). The scale bars represent 50μm. Data represent the means with SD of 6 to 12 animals per group. The analysis was performed by ANOVA, in which the post hoc Tukey test was used for multiple comparisons. Significant differences are presented.

2.1 Supplementary Figure S3

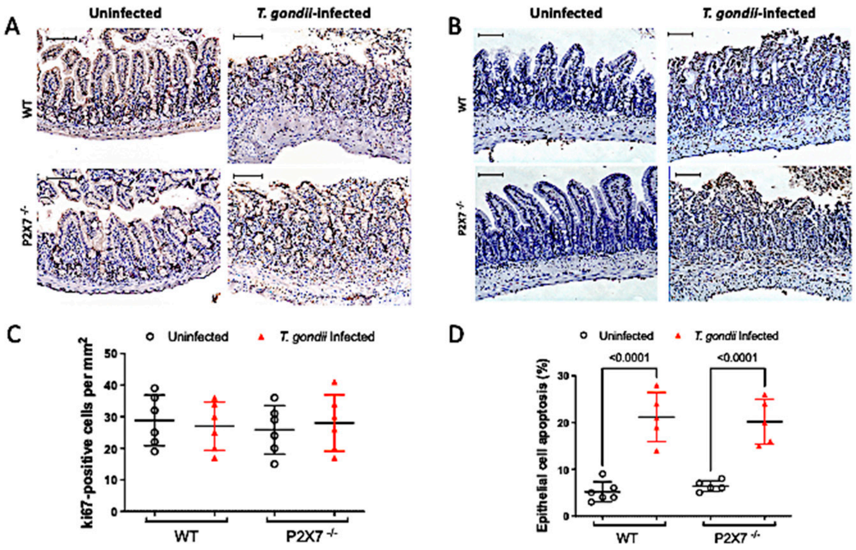

Supplementary Figure S3. *T. gondii* infection is characterized by intense ileitis with marked cell loss. Regenerative mechanisms may not be sufficient in the acute phase of the infection, and the expression of the ki67-proliferation cell marker did not show any difference among the groups (A, C). Nevertheless, apoptotic cells detected using a TUNEL assay showed a marked increase in *T. gondii*-induced mice, but no difference was observed regarding the P2X7 expression (B, D). Values are the means with SD of 5 to 6 animals per group. The analysis was performed by ANOVA, in which the post hoc Tukey test was used for multiple comparisons. Significant differences are presented.

2.1 Supplementary Figure S4

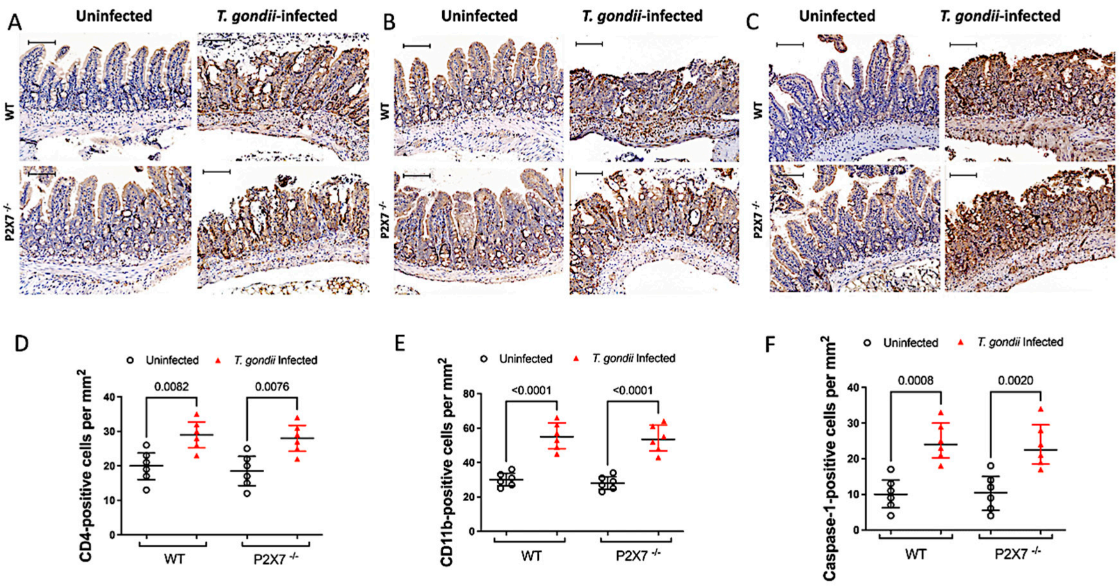

Supplementary Figure S4. *T. gondii*-induced ileitis is characterized by a marked inflammatory cell accumulation and activation in the intestinal mucosa. The expression of CD4- and CD11b-positive cells, representing mononuclear immune-active cells, and caspase-1, a critical enzyme for activating IL-1 beta, for example (A,B,C), were significantly increased in infected mice, but no difference was detected regarding the expression of the P2X7 (D, E, F). Values are the means with SD of 5 to 6 animals per group. The analysis was performed by ANOVA, in which the post hoc Tukey test was used for multiple comparisons. Significant differences are presented.

2.1 Supplementary Figure S5

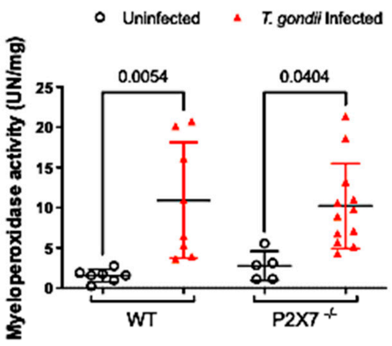

Supplementary Figure S5. *T. gondii*-induced ileitis is characterized by increased myeloperoxidase activity (MPO) in the affected tissue. However, no significant difference was observed regarding the expression of the P2X7. Values are the means with SD of 5 to 12 animals per group. The analysis was performed by ANOVA, in which the post hoc Tukey test was used for multiple comparisons. All statistical values are presented.

2.1 Supplementary Figure S6

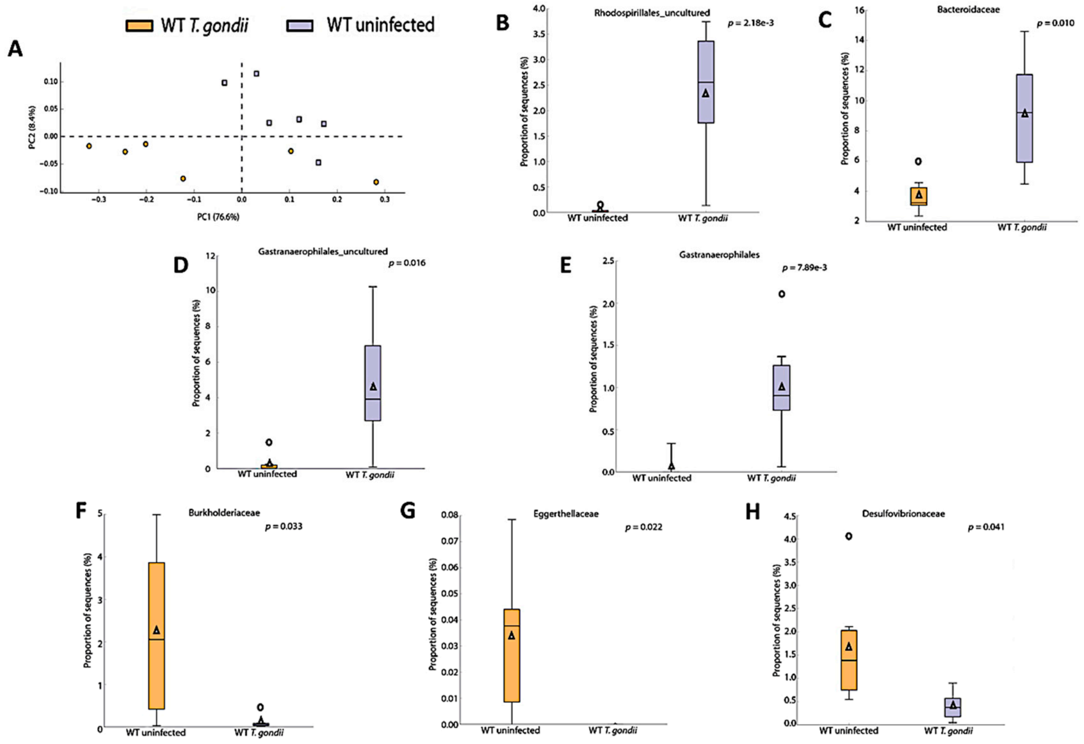

Supplementary Figure S6. *T. gondii*-induced ileitis is associated with changes in the gut microbiota of the WT mice. The Principal Component Analysis (PCA) shows the differences between the WT *T. gondii* and WT uninfected groups (A). The box plots show the distribution in the proportion of operational taxonomic units (%). WT *T. gondii* infection induced an increase in Rhodospirillales (B), Bacteroidaceae (C), and Gastranaerophilales (D, E), and a reduction in Burkholderiaceae (F), Eggerthellaceae (G), and Desulfovibrionaceae (H), compared with WT uninfected. The boxes represent the interquartile ranges (IQR) of 6 to 7 animals per group. The median value is shown as a line within the box and the mean as a triangle. Whiskers extend to the most extreme value. Outliers are shown as a circle. The *p*-value analysis was performed by Kruskal-Wallis, in which multiple comparisons were carried out using Dunn's post hoc test. Considering the *p*-value of 0.05, the variations shown are significant.

2.1 Supplementary Figure S7

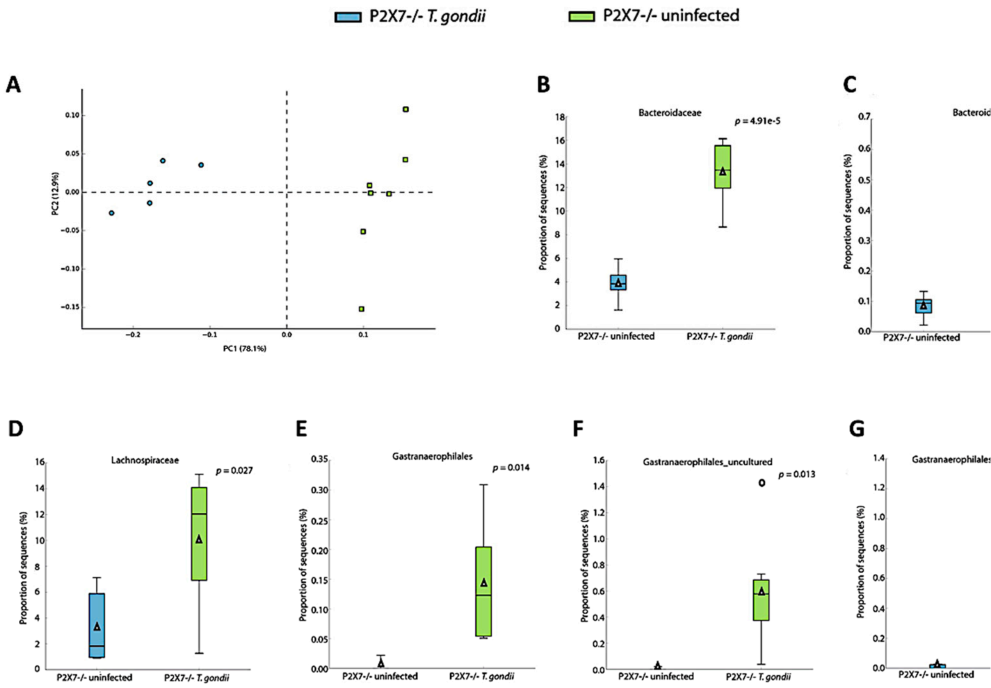

Supplementary Figure S7. *T. gondii*-induced ileitis is associated with changes in the gut microbiota of the P2X7-/- mice. The Principal Component Analysis (PCA) shows a clear separation between the P2X7-/- *T. gondii* and P2X7-/- uninfected groups (A). The box plots show the distribution in the proportion of operational taxonomic units (%). P2X7-/- T. gondii infection induced increases in Bacteroidaceae (B), Bacteroidales (C), Lachnospiraceae (D), and Gastranaerophilales (E, F, G), compared with P2X7-/- uninfected. The boxes represent the interquartile ranges (IQR) of 6 to 7 animals per group. The median value is shown as a line within the box and the mean as a triangle. Whiskers extend to the most extreme value. Outliers are shown as a circle. The *p*-value analysis was performed by Kruskal-Wallis, in which multiple comparisons were carried out using Dunn's post hoc test. Considering the *p*-value of 0.05, the variations shown are significant.

2.1 Supplementary Figure S8

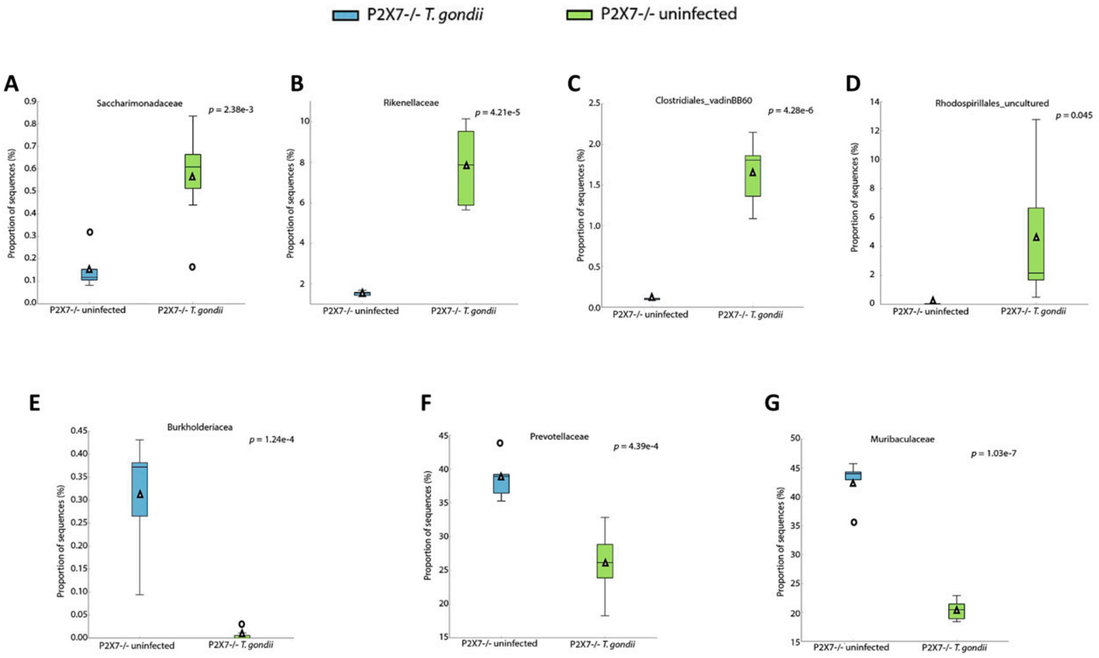

Supplementary Figure S8. *T. gondii*-induced ileitis is associated with changes in the gut microbiota of the P2X7-/- mice. The box plots show the distribution in the proportion of operational taxonomic units (%). P2X7-/- *T. gondii* infection induced increases in Saccharimonadaceae (A), Rikenellaceae (B), Clostridiales vadin (C), Rhodospirillales (D), and reductions in Burkholderiacea (E), Prevotellaceae (F), and Muribaculaceae (G) compared with P2X7-/- uninfected. The boxes represent the interquartile ranges (IQR) of 6 to 7 animals per group. The median value is shown as a line within the box and the mean as a triangle. Whiskers extend to the most extreme value. Outliers are shown as a circle. The *p*-value analysis was performed by Kruskal-Wallis, in which multiple comparisons were carried out using Dunn's post hoc test. Considering the *p*-value of 0.05, the variations shown are significant.

2.1 Supplementary Figure S9

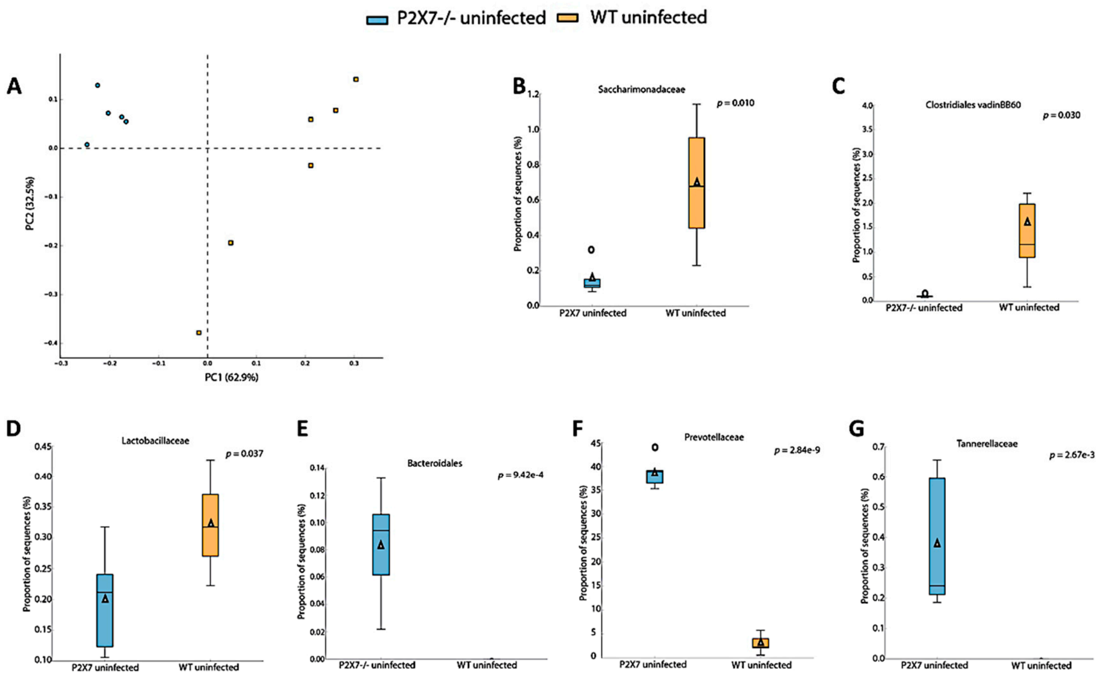

Supplementary Figure S9. The P2X7 receptor modulates the gut microbiota in mice. The Principal Component Analysis (PCA) shows a clear separation between P2X7-/- uninfected and WT uninfected mice (A). The box plots show the distribution in the proportion of operational taxonomic units (%). P2X7-/- uninfected is associated with a reduction in Saccharimonadaceae (B), Clostridiales vadin (C), and Lactobacillaceae (D), and increases in Bacteroidales (E), Prevotellaceae (F), and Tannerelaceae (G), compared with WT uninfected. The boxes indicate the interquartile ranges (IQR) of 6 to 7 animals per group. The median value is shown as a line within the box and the mean as a triangle. Whiskers extend to the most extreme value. Outliers are shown as a circle. The *p*-value analysis was performed by Kruskal-Wallis, in which multiple comparisons were carried out using Dunn's post hoc test. Considering the *p*-value of 0.05, the variations shown are significant.

2.1 Supplementary Figure S10

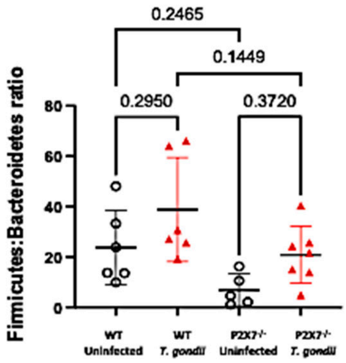

Supplementary Figure S10. Effect of P2X7 on microbiota composition considering the Firmicutes: Bacteroidetes ratio of fecal samples in *T. gondii*-induced ileitis. No significant changes were detected among the experimental groups. Values are the means with SD of 5 to 7 animals per group. The analysis was performed by ANOVA, in which the post hoc Tukey test was used for multiple comparisons. All statistical values are presented.

3. References

1 Klindworth A, Pruesse E, Schweer T, Peplies J, Quast C, Horn M, Glockner FO. Evaluation of general 16S ribosomal RNA gene PCR primers for classical and next-generation sequencing-based diversity studies. *Nucleic Acids Res* 2013; **41**(1): e1 [PMID: 22933715 PMCID: PMC3592464 DOI: 10.1093/nar/gks808]

2 Ewels P, Magnusson M, Lundin S, Kaller M. MultiQC: summarize analysis results for multiple tools and samples in a single report. *Bioinformatics* 2016; **32**(19): 3047-3048 [PMID: 27312411 PMCID: PMC5039924 DOI: 10.1093/bioinformatics/btw354]

3 Katoh K, Misawa K, Kuma K, Miyata T. MAFFT: a novel method for rapid multiple sequence alignment based on fast Fourier transform. *Nucleic Acids Res* 2002; **30**(14): 3059-3066 [PMID: 12136088 PMCID: PMC135756 DOI: 10.1093/nar/gkf436]

4 Price MN, Dehal PS, Arkin AP. FastTree 2--approximately maximum-likelihood trees for large alignments. *PLoS One* 2010; **5**(3): e9490 [PMID: 20224823 PMCID: PMC2835736 DOI: 10.1371/journal.pone.0009490]

5 Pruesse E, Quast C, Knittel K, Fuchs BM, Ludwig W, Peplies J, Glockner FO. SILVA: a comprehensive online resource for quality checked and aligned ribosomal RNA sequence data compatible with ARB. *Nucleic Acids Res* 2007; **35**(21): 7188-7196 [PMID: 17947321 PMCID: PMC2175337 DOI: 10.1093/nar/gkm864]

6 Faith DP. Phylogenetic pattern and the quantification of organismal biodiversity. *Philos Trans R Soc Lond B Biol Sci* 1994; **345**(1311): 45-58 [PMID: 7972354 DOI: 10.1098/rstb.1994.0085]

7 Lozupone CA, Hamady M, Kelley ST, Knight R. Quantitative and qualitative beta diversity measures lead to different insights into factors that structure microbial communities. *Appl Environ Microbiol* 2007; **73**(5): 1576-1585 [PMID: 17220268 PMCID: PMC1828774 DOI: 10.1128/AEM.01996-06]
